# Male reproductive ageing: a radical road to ruin

**DOI:** 10.1093/humrep/dead157

**Published:** 2023-08-11

**Authors:** R John Aitken

**Affiliations:** Priority Research Centre for Reproductive Science, Discipline of Biological Sciences, School of Environmental and Life Sciences, College of Engineering Science and Environment, University of Newcastle, Callaghan, NSW, Australia; Hunter Medical Research Institute, New Lambton Heights, NSW, Australia

**Keywords:** male ageing, declining fertility, declining testosterone, *de novo* mutations, sperm DNA damage

## Abstract

In modern post-transition societies, we are reproducing later and living longer. While the impact of age on female reproductive function has been well studied, much less is known about the intersection of age and male reproduction. Our current understanding is that advancing age brings forth a progressive decline in male fertility accompanied by a reduction in circulating testosterone levels and the appearance of age-dependent reproductive pathologies including benign prostatic hypertrophy and erectile dysfunction. Paternal ageing is also associated with a profound increase in sperm DNA damage, the appearance of multiple epigenetic changes in the germ line and an elevated mutational load in the offspring. The net result of such changes is an increase in the disease burden carried by the progeny of ageing males, including dominant genetic diseases such as Apert syndrome and achondroplasia, as well as neuropsychiatric conditions including autism and spontaneous schizophrenia. The genetic basis of these age-related effects appears to involve two fundamental mechanisms. The first is a positive selection mechanism whereby stem cells containing mutations in a mitogen-activated protein kinase pathway gain a selective advantage over their non-mutant counterparts and exhibit significant clonal expansion with the passage of time. The second is dependent on an age-dependent increase in oxidative stress which impairs the steroidogenic capacity of the Leydig cells, disrupts the ability of Sertoli cells to support the normal differentiation of germ cells, and disrupts the functional and genetic integrity of spermatozoa. Given the central importance of oxidative stress in defining the impact of chronological age on male reproduction, there may be a role for antioxidants in the clinical management of this process. While animal studies are supportive of this strategy, carefully designed clinical trials are now needed if we are to realize the therapeutic potential of this approach in a clinical context.

## Graphical Abstract

**Graphical Abstract:**
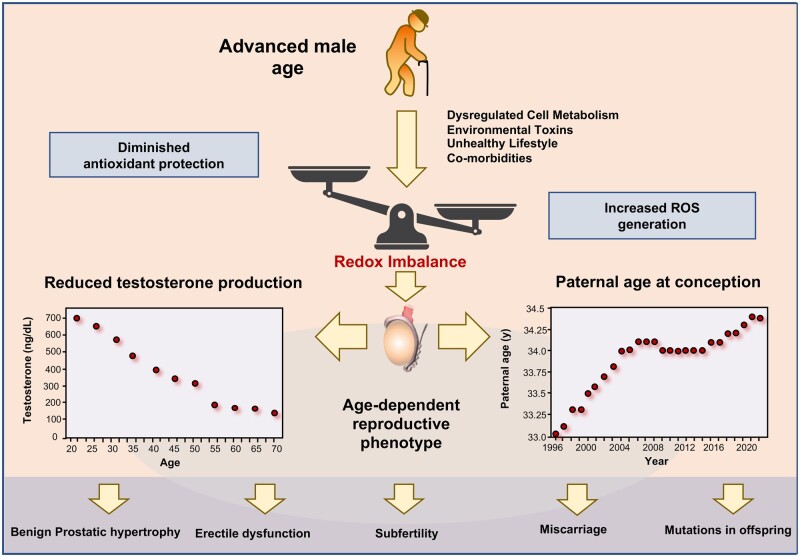
**Paternal ageing leads to redox imbalance in reproductive tissues and pathologies that impact both testosterone production and the functional and genetic integrity of the germ line.** ROS, reactive oxygen species.

## Introduction

An inevitable consequence of increasing socioeconomic development is that societies experience a demographic transition, whereby family sizes decrease in response to a reduction in infant and childhood mortality (Aitken, 2022a). Societies that have been through this transition are characterized by low total fertility rates and delayed parenthood, such that the age at which women have their first child is around 30 years in most advanced economies (Aitken, 2022a,b). The impact of advanced maternal age on fertility and offspring health are well documented (Balasch and Gratacós, 2012; Johnson et al., 2012). However, the consequences of advanced paternal age on reproductive efficiency and offspring health have not been so thoroughly considered. This is a critical issue for contemporary society where the average age of fathers at the moment of conception has been steadily increasing for both natural and assisted conceptions (Bray *et al.*, 2006; Lawson and Fletcher, 2014; Brandt *et al.*, 2019). Our current understanding is that increasing paternal age is associated with a range of issues affecting fertility, the health and wellbeing of the offspring, and the functionality of the reproductive system in general (Lawson and Fletcher, 2014; Aitken *et al.*, 2020; Aitken, 2022c). In light of its significance, this review examines the impact of age on the male reproductive system and considers the nature of the underlying mechanisms.

## Age, fertility, and the semen profile

Age is the enemy of both male and female fertility. However, whereas female fecundity falls precipitously between the age of 35 and 40, in men the decline is much more gradual. Using time to conception as a measure of fertility in untreated couples, Ford *et al.* (2000) found that the adjusted odds ratio for natural conception in ≤12 months is halved in men over 35 compared to their <25 year old counterparts. Similarly, in the case of IVF and IUI cycles, both implantation and pregnancy rates have been found to decrease in relation to increased paternal age, while positive correlations have been observed with the risk of miscarriage (Mathieu *et al.*, 1995; Dunson *et al.*, 2002; Belloc *et al.*, 2008, 2014; Wu *et al.*, 2015). Even more convincingly, when maternal age is controlled by the use of donor oocytes in ART cycles, impacts of paternal age on fertilization success, embryo quality, and live birth rates have all been observed (Setti *et al.*, 2020; Vogiatzi *et al.*, 2022; Begon *et al.*, 2023; Coban *et al.*, 2023). Despite some inconsistency in the literature (Beguería *et al.*, 2014), the general conclusion drawn from such studies is that paternal age impacts both the fertilizing capacity of the spermatozoa and their ability to support normal embryonic development to term. How does chronological age achieve this effect?

The notion that the conventional parameters of semen quality (count, motility, and morphology) deteriorate with age is controversial, since such changes have been observed in some datasets but not others (Plas *et al.*, 2000; Eskenazi *et al.*, 2003; Zhu *et al.*, 2011; Oliveira *et al.*, 2014; Johnson *et al.*, 2015). Such contradictory data may well be a reflection of the confounding impact of changes in ejaculatory frequency that are known to accompany male ageing (Lee *et al.*, 2016). These discrepancies may also reflect differences between studies in the depth of the fundamental semen analysis. Thus, when CASA is used to provide a detailed objective analysis of sperm movement characteristics, then significant declines in the quality of sperm motility with paternal age are consistently observed (Sloter *et al.*, 2006; Fréour *et al.*, 2012; Verón *et al.*, 2018). It is also possible that ageing affects aspects of sperm function that are not accurately reflected in a simple conventional semen profile. For example, significant differences in sperm ultrastructure (Baccetti *et al.*, 1984) as well as the sperm proteome (Liu *et al.*, 2015; Bastos *et al.*, 2017; Guo *et al.*, 2023) have been noted with advanced male age, affecting structures, and proteins potentially involved in both sperm motility and fertilization. Furthermore, an integrated analysis of the sperm metabolome and proteome identified differential energy metabolism and oxidative stress related proteins as being impacted by paternal age (Guo *et al.*, 2023). This is a particularly important finding since it resonates with additional data suggesting that oxidative stress is critically involved in the age-dependent decline of male fertility, as would be anticipated by the free radical theory of ageing (Harman 1956).

## Age, oxidative stress, and sperm DNA damage

Ageing is associated with a significant reduction in the ability of the body to prevent, detect and repair cellular damage induced by free radicals (largely reactive species of oxygen and nitrogen as well as their secondary metabolites), generated primarily as a result of aerobic metabolism (Wickens, 2001). This process leads to an accumulation of damage to proteins, lipids, and nucleic acids that collectively compromise the normal physiological functions of cells, tissues, and organs to the point that oxidative stress is induced and the ageing process becomes manifest. While this theory focuses on free radical-mediated damage, in truth, the ageing process can also be precipitated by a variety of reactive oxygen species (ROS) that are not free radicals, including the damaging oxidant, hydrogen peroxide. Every physiological system is vulnerable to oxidative attack, but the brain and the reproductive system appear to be particularly susceptible to this form of pathological insult. As a consequence, oxidative stress has been associated with a wide range of age-related, neurodegenerative diseases (including Alzheimer’s, Parkinson’s, amyotrophic lateral sclerosis, and Huntington’s disease) as well as both male and female infertility (Aitken *et al.*, 2022a; Shadfar *et al.*, 2023).

In terms of male infertility, oxidative stress has been recognized as an important cause of this condition for more than 35 years since defective sperm function was found to be associated with the enhanced generation of ROS in the ejaculate (Aitken and Clarkson, 1987; Alvarez *et al.*, 1987). The excessive generation of ROS has been linked to seminal leukocyte contamination (Aitken *et al.*, 1995) while, in the germ line, the dysregulation of electron transport in the sperm mitochondria as well as increases in the activities of NADPH—and/or amino acid—oxidases have all been cited as potential contributors to the oxidative stress that culminates in male infertility (Aitken and Baker, 2020; Aitken *et al.*, 2022a,b). A state of oxidative stress may also be induced by a lack of antioxidant protection, since many studies have indicated that the antioxidant protection provided by seminal plasma or the spermatozoa themselves is frequently compromised in cases of male infertility (Benedetti *et al.*, 2012; Fernandez and O'Flaherty, 2018; Gupta *et al.*, 2021; O'Flaherty and Scarlata, 2022).

In light of the above, redox imbalance is a critical feature of male infertility and one that is profoundly influenced by age (Aitken and Baker, 2020; Guo *et al.*, 2023). In animal models, there is a great deal of evidence to suggest that ageing is associated with oxidative stress in the male germ line. For example, functional deletion of two important antioxidant enzymes, thioredoxin 2 and 3 (*Txndc2, Txndc3*) in male mice leads to a series of age-dependent pathological changes in the spermatozoa brought on by the resulting oxidative stress, including accelerated sperm motility loss, high rates of DNA damage, increased ROS generation, enhanced formation of lipid aldehyde-protein adducts, and impaired protamination of the sperm chromatin (Smith *et al.*, 2013). Similarly, the *Prdx6*−/− knock out mouse exhibits an age-dependent impairment of sperm motility and maturation, increasing sperm DNA fragmentation/oxidation while decreasing DNA compaction and protamination (Ozkosem *et al.*, 2015). Depletion of a major SOD gene (*Sod1*−/−) is also associated with an age-dependent increase in sperm oxidative DNA and lipid peroxidation (Noblanc *et al.*, 2020). In a human context, an age-dependent increase in sperm lipid peroxidation has been recorded (Vaughan *et al.*, 2020) and there are several powerful studies indicating that paternal age has a major impact on the levels of DNA integrity exhibited by the spermatozoa (Rosiak-Gill *et al.*, 2019; Evenson *et al.*, 2020).

Given this background, it is clear that as males age their germ line comes under increasing oxidative attack leading to lipid peroxidation, a loss of key DNA repair pathways, and a significant increase of DNA fragmentation (Almeida *et al.*, 2017; Nguyen-Powanda and Robaire, 2021). One of the reasons for this progressive increase in oxidative damage to the germ line may be a reduction in the levels of antioxidant protection afforded to these cells with advanced age (Tarín *et al.*, 1998; Nago *et al.*, 2021). A formal analysis of antioxidant levels in human seminal plasma as a function of male age would be helpful in this context, and is long overdue.

## Ageing and telomere length

Since guanine residues are particularly vulnerable to oxidative attack and telomeres are guanine rich structures ((TTAGGG)n), it might be anticipated that they would be vulnerable to oxidative damage during the ageing process. Existing data clearly suggest that this is the case, although the consequences of such oxidative stress appear to depend on the severity of the attack. Thus, if the oxidative insult is acute and severe, as might be the case in reproductive-age patients suffering from infertility associated with conditions such as varicocele or acute reproductive tract infection, then the net result will be DNA fragmentation and telomere shortening in the spermatozoa (Darmishonnejad *et al.*, 2019, 2020; Aitken *et al.*, 2020; Moazamian *et al.*, 2022). However, if the oxidative stress is more prolonged and progressive, as occurs with ageing, then a completely different picture emerges. Because the male germ line is one of the only adult cell types to express telomerase activity (Zalenskaya and Zalensky, 2002), it is able to mount an adaptive response to any free radical-mediated attack on telomere integrity. Consequently, as men age and their testes become exposed to gradually increasing levels of oxidative stress, telomerase responds and telomere length in the germ line significantly increases. This biphasic relationship between oxidative stress and telomere length has also been recorded in the (non-aged) infertile male population, in whom severe oxidative stress is linked with sperm telomere shortening while mild oxidative stress results in telomere lengthening (Mishra *et al.*, 2016). These relationships are important because they may have an important bearing on the health and wellbeing of the progeny, given that telomere length is a paternally inherited trait; the older the father, the longer the telomeres in his spermatozoa and the longer the telomeres in his offspring (Eisenberg *et al.*, 2012). Moreover, this intriguing relationship is additive across generations. Therefore, if your grandfather was old when your father was conceived and your father was old when you were conceived, you are likely to have very long telomeres. It might be imagined that this would be a cause for celebration because telomere length is proportional to longevity (Kappei and Londoño-Vallejo, 2008). However, life might not be that simple. The presence of long telomeres extends the number of cell cycles that are possible within any given cell lineage, before the Hayflick limit is reached and apoptosis is induced. The extended number of replications associated with long telomeres may therefore increase the risk of mutation due to a replication error and/or prolong the exposure of a given cell lineage to pathological environmental toxins. Clearly, there is still a great deal that we still do not understand about cell replication and its relation to conditions such as cancer. However, recent data suggest that long telomeres may be associated with an increased risk for the appearance of melanoma, lung adenocarcinoma, and a variety of other cancers (McNally *et al.*, 2019). Thus, the long telomeres acquired as a result of advanced paternal age at the moment of conception, might be yet another example of how age impacts not just the reproductive health of males, but also the health and wellbeing of their offspring.

## Ageing and offspring health

We have been aware of the powerful impact of paternal ageing on offspring health since Weinberg (1912) noted the relationship that exists between the incidence of spontaneous achondroplasia and the birth sequence of children within a family, with the last-born child being the most susceptible. Subsequently, Penrose (1955) demonstrated that this relationship reflected the importance of paternal age in the aetiology of dominant genetic disease. We have subsequently become aware that many dominant genetic diseases within our species share the same profound relationship with paternal age including a number that present as single gene defects, such as Apert syndrome, Crouzon syndrome, Pfeiffer syndrome, achondroplasia, thanatophoric dysplasia, multiple endocrine neoplasia type 2, and multiple endocrine neoplasia type 2b (Aitken, 2022c). Conditions such as achondroplasia and Apert syndrome, are known to be induced by single point mutations in fibroblast growth factor receptors (FGFRs): FGFR2, in the case of Apert syndrome, and FGFR3, in the case of achondroplasia (Crow, 2000). Traditionally the explanation for this relationship between paternal age and the appearance FGFR mutations has been ‘replication error’ (Haldane, 1935). According to this hypothesis, the number of rounds of replication that the male germ line undergoes, increases as a linear function of paternal age such that fathers at the age of 20 and 60 years, would generate spermatozoa after 199 and 1119 rounds of replication, respectively (Goldmann *et al.*, 2019). Since every round of cell division carries a risk of replication error, it is not surprising that both the number of germ line replications and the mutational load carried by children increase linearly with paternal age (Kong *et al.*, 2012), suggesting a causative association. However, the chances that such specific mutational changes in the *FGFR* genes might occur randomly in a genome containing 3.2 billion base pairs are infinitesimally small and therefore have no relationship at all to the observed incidence of the conditions which, in the case of achondroplasia, can be as high as 35 per 100 000 in North Africa and the Middle East and around 4.6 per 100 000 worldwide. The answer to this conundrum can be found in the ‘selfish spermatogonial selection’ model which suggests that the FGFR mutations responsible for these dominant genetic conditions confer upon the mutated germ cells a selective advantage that allows their numbers to increase dramatically, in an oncogenic-like process, resulting in clonal expansion of the mutated cells, in locations along the seminiferous tubule (Goriely *et al.*, 2003, 2013; Goriely and Wilkie, 2012). In accordance with this model, histological sections through the testis of ageing males have revealed the predicted clusters of the mutated germ cells, each one of which is capable of differentiating into a spermatozoon that will result in the emergence of genetic disease in the progeny (Goriely *et al.*, 2003; Maher *et al.*, 2018). This hypothesis may also be applicable to other dominant genetic diseases caused by paternal age, including multiple endocrine neoplasia type 2b, where the causative mutation can be found in the *RET* (rearranged during transfection) proto-oncogene (Choi *et al.*, 2012), Noonan syndrome, which is driven by a mutation in the *PTPN11* gene (encoding protein tyrosine phosphatase non-receptor type 11) (Liao and Mehta, 2019), and Costello syndrome, which involves a mutation in the *HRAS* proto-oncogene(Harvey rat sarcoma viral oncogene homolog, GTPase) (Nagai *et al.*, 2022). Other rare conditions, such as the so-called RASopathies, are also associated with mutations in genes encoding components of the RAS (reticular activating system)/rapidly accelerated fibrosarcoma/mitogen-activated protein kinases signal transduction pathway, including LEOPARD syndrome and hereditary gingival fibromatosis (Tidyman and Rauen, 2009; Maher *et al.*, 2018). Overall, these studies indicate that age is associated with a range of spontaneous mutations in male germ cells that confer upon their descendant cells a selective advantage, thereby permitting their clonal expansion. This ‘selfish spermatogonial selection’ model therefore proposes a powerful mechanism by which extremely rare spontaneous mutations in the spermatogonial stem cell population may be responsible for a range of well-established autosomal dominant conditions in humans. However, this is not the whole story.

## Paternal ageing and *de novo* mutations

**Figure 1.**
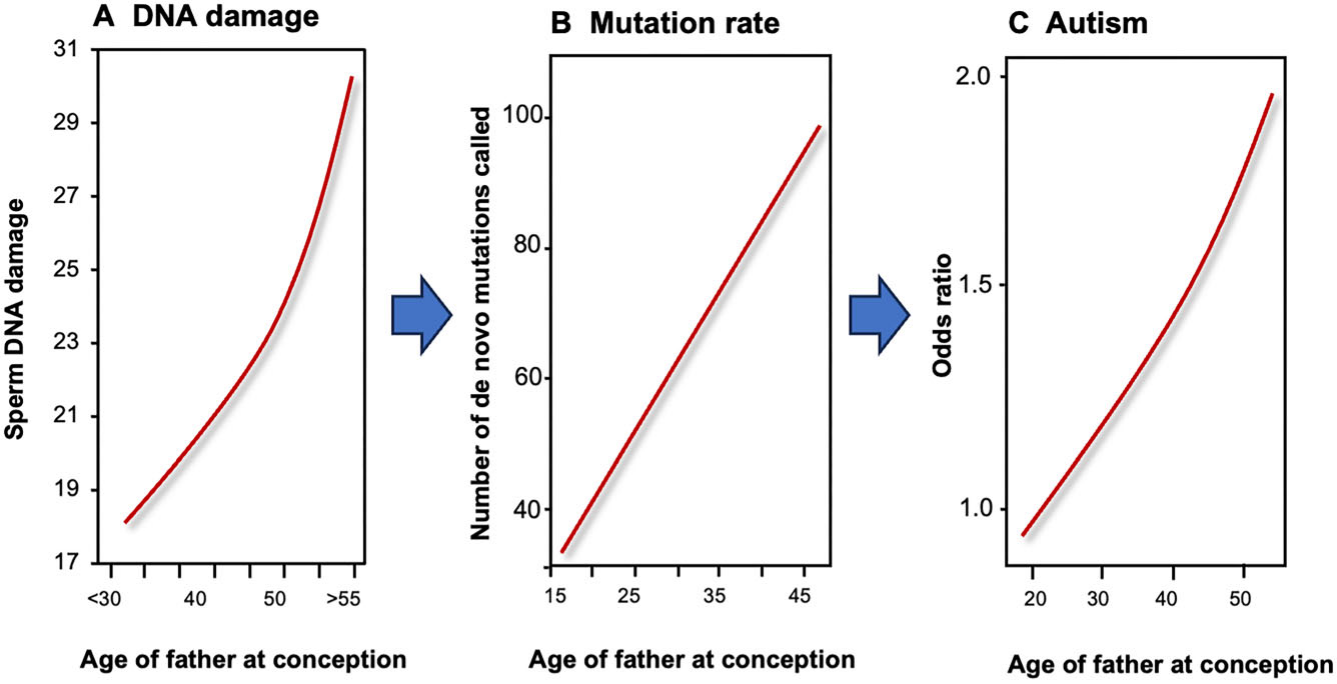
**General relationships between paternal age, sperm DNA damage, the mutational load carried by the embryos, and the incidence of neuropsychiatric conditions such as autism in the offspring.** (**A**) As males age, the levels of DNA damage in their spermatozoa increase dramatically. (**B**) In parallel, the incidence of spontaneous mutations increases in the offspring, a component of which may involve aberrant sperm DNA damage repair following fertilization as predicted by the post-meiotic oocyte collusion hypothesis (Aitken, 2022c). (**C**) As the incidence of spontaneous mutations increases, so does the appearance of pathologies in the offspring, including neuropsychiatric conditions such as autism. These changes are consistent with, but do not prove, a chain of causal relationships between paternal age, increased DNA damage in the spermatozoa, an enhanced mutational load in the embryos and the consequential appearance of pathologies in the offspring that have a strong genetic basis, such as autism (Wyrobek *et al.*, 2006; Croen *et al.*, 2007; Sasanfar *et al.*, 2010; Kong *et al.*, 2012; Aitken *et al.*, 2020, 2022c; Vaughan *et al.*, 2020).

Around 75% of all *de novo* mutations in our species occur in the male germ line and are strongly associated with paternal age (Gao *et al.*, 2019). These mutations cover a wide range of genetic targets and cannot all have arisen as a result of a selective advantage that allows the clonal expansion of mutated stem cells; some other explanation must be entertained. In this context, a plausible hypothesis is that the low, steady rate of spontaneous mutations arising as a result of replication errors associated with paternal age (Moore *et al.*, 2021) can be augmented by mutations that arise as a result of defective repair of the DNA damage arising in the male germ line. As indicated above, we already know that DNA damage in human spermatozoa increases as an exponential function of paternal age (Evenson *et al.*, 2020). A significant proportion of these DNA-damaged cells must retain the capacity for fertilization because we see correlations between paternal age and both the quality of blastocysts created during ART (Donatti *et al.*, 2023) as well as the incidence of spontaneous early pregnancy loss (Brandt *et al.*, 2022). Once fertilization has occurred, the oocyte will have a brief period time to repair the DNA damage brought in by the fertilizing spermatozoon before S-phase of the first mitotic division is activated. If this repair process is inaccurate or deficient, then a mutation may arise that could potentially impact the health and wellbeing of the offspring (Fig. 1). This mechanism for the creation of *de novo* mutations is known as the ‘post-meiotic oocyte collusion hypothesis’ (Aitken, 2022c) and is in keeping with the finding that the earliest stages of embryogenesis are extremely mutagenic (Goldmann *et al.*, 2019). An important feature of this hypothesis is that mutations are held to arise through the combined action of male and female germ lines. The male is responsible for creating pre-mutational lesions in the paternal DNA, while the female is responsible for the effective repair of this damage following fertilization. Importantly, advanced age will impact both sides of this equation, promoting DNA damage in the spermatozoa on the one hand while impairing the DNA repair capacity of the oocyte on the other (Evenson *et al.*, 2020; Horta *et al.*, 2020; Punjabi *et al.*, 2022; Aitken, 2022c). As a consequence of these associations, there is an additive effect on pregnancy and live birth rates when both partners are of an advanced age in ART cycles (McPherson *et al.*, 2018; Setti *et al.*, 2021).

If the sperm DNA damage seen in men as a function of their age is oxidatively induced then it becomes important to know which region of the sperm genome is the most vulnerable to oxidative attack. An analysis of this issue has revealed that while the sex chromosomes are, to some extent, resistant to oxidative stress, the autosomes are all vulnerable to such attacks, particularly chromosome 15 (Xavier *et al.*, 2019). According to this analysis, the areas of vulnerability to oxidative stress within the sperm chromatin are the interlinker regions that lie between the protamine-dominated toroids and are attached to the nuclear matrix. An analysis of the distribution of oxidized bases across chromosomes revealed that the q13–14 region of chromosome 15 was particularly vulnerable to oxidative attack (Xavier *et al.*, 2019). Interestingly, mutations in this area of the genome are known to be associated with a variety of neuropathological conditions including autism spectrum disorders, susceptibility to epilepsy, schizophrenia, bipolar disorder, Marfan syndrome, congenital heart defects, and obsessive-compulsive disorder (Aitken *et al.*, 2020; Aitken, 2022c). Furthermore, many of these conditions are known to be correlated with paternal age at the time of conception (Malaspina *et al.*, 2002; Croen *et al.*, 2007; Frans *et al.*, 2008). So, a pattern of cause and effect appears to exist whereby advanced paternal age precipitates oxidative stress in the germ line, leading to oxidative DNA damage in all areas of the genome, but with a hot-spot on chromosome 15. Oxidative damage to the genome may then lead to the appearance of mutations in the offspring, possibly as a consequence of defective/deficient DNA damage repair in the oocyte. These mutations then increase the risk of genetic disease in the progeny, with particular emphasis on a range of neuropsychiatric and behavioural disorders that are known to be correlated with paternal age (Zweifel and Woodward, 2022).

## Paternal ageing and epigenetic change

While this analysis has focused on age-dependent genetic changes that occur within the genome of the male germ line, we should not ignore the potential impact of an equivalent increase is epigenetic changes associated with advanced paternal age (Yatsenko and Turek, 2018). Hundreds of DNA methylation changes are evident in the spermatozoa of ageing males, many of which are present in the CpG regions that regulate the expression of key genes governing neurological, psychiatric and behavioural disorders, including conditions such as spontaneous schizophrenia, bipolar disease, mood disorders and autism, that are known to be increased in the offspring of ageing fathers (Milekic *et al.*, 2015; Yatsenko and Turek, 2018). Importantly, recent data have confirmed that epigenetic changes in spermatozoa are associated with the appearance of autism-spectrum changes in the offspring (Feinberg *et al.*, 2023) and could be a key mechanism by which paternal age impacts the health and wellbeing of children. The molecular mechanisms underpinning these epigenetic changes in the male germ line are unknown but could be yet another reflection of the oxidative stress that characterizes male reproductive ageing (Menezo *et al.*, 2016). Oxidative stress is capable of attacking several epigenetic elements in spermatozoa including DNA methylation, non-coding RNA species and changes to both the quantity and methylation status of histones incorporated into the sperm genome during spermatogenesis (Sharma *et al.*, 2019; Liang *et al.*, 2021). The impact of age-related oxidative stress on the epigenetic status of the male gamete, and the resultant implications for the health and wellbeing of the offspring, is an emerging area of research that will clearly shape our future understanding of paternal impacts on development (Ashapkin *et al.*, 2023).

## Paternal ageing, testosterone, and oxidative stress

The germ line is not the only aspect of the male reproductive system that is negatively impacted with age; the somatic cells of the testes (including the Sertoli cells, Leydig cells, peritubular myoid cells, macrophages, and endothelial cells) also exhibit molecular signs of damage in ageing men (Nie *et al.*, 2022). These regressive changes underpin the reported changes in spermatogenesis with age, as well as changes in the endocrine functions of the testes, particularly testosterone secretion (Haji *et al.*, 1994; Bohring and Krause, 2003; Dong *et al.*, 2022).

For both Sertoli and Leydig cells, there is evidence that the impact of ageing on the functionality of these cells is mediated by oxidative stress. For example, glutathione depletion is known to result in a reduction in the ability of Leydig cells to generate testosterone levels via mechanisms that can be reversed by the presence of antioxidants (Chen *et al.*, 2008) or the activation of antioxidant pathways mediated by Sirt1 (sirtuin 1) and/or Nrf2 (nuclear factor erythroid 2) (Chung *et al.*, 2021) both of which become down-regulated with age (Kopalli *et al.*, 2019; Zhao *et al.*, 2021). Antioxidants, or compounds that enhance endogenous antioxidant activity, such as the co-factor nicotinamide, have also been shown to enhance testosterone production in ageing rats and preserve the structural integrity of the testes (Hacioglu *et al.*, 2021; Al-Shaikh, 2022). Other putative antioxidants such as Korean red ginseng (Kopalli *et al.*, 2017), Cordycepin (Kopalli *et al.*, 2019), Gigantol (Basque *et al.*, 2021), and vitamin C (Heidari *et al.*, 2023) have similar effects. The source of oxidative stress in ageing Leydig cells is thought to be mitochondrial (Chen *et al.*, 2001; Miquel, 2002; El Kattawy *et al.*, 2022), while the target is thought to be the hormone-sensitive mitochondrial cholesterol transfer step of steroidogenesis (Diemer *et al.*, 2003).

## Oxidative stress as the common denominator

It is clear from the data presented above that oxidative stress is powerful driver of the ageing process in general and the age-dependent decline in male reproductive function, in particular. The impact of age on testicular function has been clearly demonstrated in genetic knock out models (Selvaratnam and Robaire, 2016) and integrated ‘-omics’ analyses (Han *et al.*, 2023). Furthermore, mitochondrial dysfunction has been repeatedly implicated as a central element of this process (Vázquez-Memije *et al.*, 2008; Amaral and Ramalho-Santos, 2009). A complex array of factors may be responsible for exacerbating the age-dependent increase in testicular ROS generation including a higher incidence and prevalence of varicoceles, a lifetime’s exposure to reproductive toxicants, lifestyle factors and co-morbidities such as obesity, diabetes, and infections, as well as the development of a pro-inflammatory state in the ageing testes (Herati *et al.*, 2017; Matzkin *et al.*, 2021). The impact of such oxidative stress is also promoted by a decline in the DNA repair capacity of the germ line (El-Domyati *et al.*, 2010; Yatsenko and Turek, 2018) as well as the reduced availability of antioxidant protection with age (Nakamura *et al.* 2010; Chen *et al.*, 2015; Kandiel and El Khawagah, 2018; Nago *et al.*, 2021).

If a reduction of antioxidant protection is an important factor in the aetiology of age-dependent male subfertility then it would seem rational to suggest that dietary antioxidant supplementation should have a beneficial effect. Data have certainly been secured from domestic animal species indicating that this is indeed the case. For example, aged roosters supplemented with co-enzyme Q10 (Sharideh *et al.*, 2020), alpha lipoic acid (Behnamifar *et al.*, 2021), or selenium (Sabzian-Melei *et al.*, 2022) demonstrate positive responses in terms of semen quality and reproductive performance. In laboratory rats, taurine or soy isoflavone supplementation has been found to postpone the age-dependent decline in testicular function, elevating the protection afforded by antioxidant enzymes, as well as reducing ROS generation, DNA fragmentation, and lipid peroxidation (Yang *et al.*, 2015; Al-Shaikh, 2022). Cerium dioxide nanoparticles, which have powerful antioxidant properties, have also been shown to ameliorate the impact of ageing on the reproductive system of rats, increasing testosterone levels and improving the quality of spermatogenesis (Kobyliak *et al.*, 2015), while an antioxidant preparation from *Ganoderma lucidum* has a similar impact on mice (Li *et al.*, 2022). The antioxidant impact of calorific restriction has also been found to increase testicular catalase, glutathione peroxidase activities, and superoxide dismutase activities in aged rats, thereby reducing lipid peroxidation and restoring spermatogenesis (Hamden *et al.*, 2008). Exogeneous testosterone therapy has also been shown to ameliorate several age-related processes in laboratory rats including age-related brain mitochondrial dysfunction (Yan *et al.*, 2021) and renal fibrosis (Zhang *et al.*, 2018) in keeping with the antioxidant impact of such treatment (Christensen *et al.*, 2023). The limited data available on the impact of antioxidants on ageing human males suggest that benefits might include a reduction in benign prostatic hypertrophy (Semenov *et al.*, 2021; Aljehani *et al.*, 2022) as well as resolution of a number of other non-communicable conditions associated with the ageing process, including weight gain, endothelial dysfunction, insulin resistance, hyperglycaemia, type 2 diabetes, and various malignancies (Leisegang *et al.*, 2021; Babcock *et al.*, 2022).

However, most clinical trials on the therapeutic benefits of antioxidants in the context of male ageing or male infertility have had serious flaws due to the inadequacy of the experimental design, low subject numbers, weak patient selection strategies, and poor product formulation (Aitken, 2021). There is really no point in conducting small-scale clinical trials with randomly selected antioxidants if they are uncontrolled and involve patients who have not been selected on the basis of oxidative stress. If powerful antioxidants are given to patients who are not suffering from such stress, the net result may be to drive them into a state of reductive stress which may be just as harmful as its oxidative counterpart (Sengupta *et al.*, 2022). The net objective of any therapeutic strategy in this area must be to restore redox balance to the system and this is not possible if the level of oxidative stress is not being continually monitored. Real progress in this field will only be made when we have raised general awareness about the importance of male reproductive ageing and have developed refined point-of-care assays for measuring oxidative stress parameters in the patient population. Only when such technologies are available can we answer the key therapeutic questions: Who should receive antioxidant therapy? When should it be given? When should it cease? and What combination of antioxidants is optimal?

## Conclusions

It is evident from clinical data available that male ageing is associated with a small but significant decline in fertility accompanied by declining testosterone levels as well as impacts on the maintenance of pregnancy and the genetic/epigenetic mutational load carried by the offspring (Aitken *et al.*, 2020; Aitken, 2022c; Begon *et al.*, 2023; Kaltsas *et al.*, 2023). While ageing is clearly an incredibly complex process, oxidative stress appears to be a core element of the underlying pathophysiology, exacerbated by a range of environmental, lifestyle and occupational factors. Given the importance of this mechanism, it is natural that the researchers should turn to the potential importance of antioxidant supplementation as a means of controlling the adverse impacts of advanced chronological age on reproductive health. In animal models, experimental data has been obtained suggesting that antioxidant supplementation can be effective in this regard (Matzkin *et al.*, 2021). However, informative clinical studies on this topic are generally sparse.

Antioxidants rich in polyphenols and lycopene have demonstrated some effectiveness against benign prostatic hypertrophy (Natali *et al.*, 2023) while several antioxidant preparations have been effective in elevating the circulating levels of testosterone (Lee *et al.*, 2020) and correcting erectile dysfunction (Meldrum *et al.*, 2012). Clearly, we are still in the early stages of such research but the potential of antioxidant therapy in the context of male ageing seems promising. With carefully designed trials (Aitken, 2021) and a focus on the ability of antioxidants to protect the mitochondria against oxidative stress, preserve the genetic and epigenetic integrity of the male germ line, and maintain circulating testosterone levels, evidenced-based therapeutic options may be available in the future. However, we are still a long way from this important goal.

## Data Availability

No new data were generated or analysed in support of this research.
